# ScAnalyzer: an image processing tool to monitor plant disease symptoms and pathogen spread in *Arabidopsis thaliana* leaves

**DOI:** 10.1186/s13007-024-01213-3

**Published:** 2024-05-31

**Authors:** Misha Paauw, Gerrit Hardeman, Nanne W. Taks, Lennart Lambalk, Jeroen A. Berg, Sebastian Pfeilmeier, Harrold A. van den Burg

**Affiliations:** 1 Molecular Plant Pathology, Faculty of Science, Swammerdam Institute for Life Sciences (SILS), University of Amsterdam, Science Park 904, Amsterdam, 1098 XH The Netherlands; 2 Technologie Centrum FNWI, Faculty of Science, University of Amsterdam, Science Park 904, Amsterdam, 1098 XH The Netherlands

**Keywords:** Plant disease, Digital phenotyping, Image processing, Bioluminescence, *Xanthomonas campestris*, *Arabidopsis thaliana*, Black rot disease

## Abstract

**Background:**

Plants are known to be infected by a wide range of pathogenic microbes. To study plant diseases caused by microbes, it is imperative to be able to monitor disease symptoms and microbial colonization in a quantitative and objective manner. In contrast to more traditional measures that use manual assignments of disease categories, image processing provides a more accurate and objective quantification of plant disease symptoms. Besides monitoring disease symptoms, computational image processing provides additional information on the spatial localization of pathogenic microbes in different plant tissues.

**Results:**

Here we report on an image analysis tool called ScAnalyzer to monitor disease symptoms and bacterial spread in *Arabidopsis thaliana* leaves. Thereto, detached leaves are assembled in a grid and scanned, which enables automated separation of individual samples. A pixel color threshold is used to segment healthy (green) from chlorotic (yellow) leaf areas. The spread of luminescence-tagged bacteria is monitored via light-sensitive films, which are processed in a similar manner as the leaf scans. We show that this tool is able to capture previously identified differences in susceptibility of the model plant *A. thaliana* to the bacterial pathogen *Xanthomonas campestris* pv. *campestris.* Moreover, we show that the ScAnalyzer pipeline provides a more detailed assessment of bacterial spread within plant leaves than previously used methods. Finally, by combining the disease symptom values with bacterial spread values from the same leaves, we show that bacterial spread precedes visual disease symptoms.

**Conclusion:**

Taken together, we present an automated script to monitor plant disease symptoms and microbial spread in *A. thaliana* leaves. The freely available software (https://github.com/MolPlantPathology/ScAnalyzer) has the potential to standardize the analysis of disease assays between different groups.

**Supplementary Information:**

The online version contains supplementary material available at 10.1186/s13007-024-01213-3.

## Background

Plants can be colonized by a plethora of pathogenic microbes, both in nature and in an agricultural context. As a consequence, disease symptoms can become visible to the naked eye, such as chlorotic or necrotic lesions on leaves. Quantifying such disease symptoms as a measure for disease severity and susceptibility is imperative in order (i) to study plant diseases including the underlying mechanisms and (ii) to facilitate data-driven decisions in plant breeding programs [1].

Traditionally, and still widely applied, macroscopic disease symptoms have been categorized in ordinal scales (‘disease indices’) ranging from little or no disease to severe disease symptoms [1]. Assessing disease severity in the context of resistance/susceptibility of individual plants is often done by human evaluations and is therefore a subjective method not readily transferred between persons and it is time consuming. Clearly, there is a need for quantifying disease symptoms in an objective manner, which is underlined by a growing number of scientific reports that describe computational pipelines that use digital images of diseased plants to quantify disease severity [2–7].

A notable example of an image-based tool to assess disease severity is PIDIQ [8], a semi-automated method to detect diseased leaf tissue of the model plant *Arabidopsis thaliana* (hereafter, Arabidopsis). PIDIQ has been used to detect differences in disease susceptibility of Arabidopsis accessions and mutants towards the bacterial pathogen *Pseudomonas syringae* pv. *tomato* DC3000 (Pst). The outcome of this Arabidopsis-Pst interaction is determined (i) by bacterial effectors that are delivered into the plant cytosol by the bacterial type III secretion system to manipulate plant processes facilitating infection, and (ii) by plant immune receptors that recognize pathogenic activity and confer resistance [8]. The pan-effectorome of Pst composed of more than 500 effectors has been screened for recognition using a worldwide collection of Arabidopsis accessions [9] using a miniaturized plant growth system based on 48-well microtiter plate with seedlings growing in the wells [10].

Besides using macroscopic disease symptoms to assess disease severity, another layer of information can be obtained by monitoring plant colonization by pathogens in a spatio-temporal manner. To visualize their presence inside plant tissue, pathogenic microbes can be genetically engineered to express reporter constructs encoding fluorescent proteins or enzymatic pathways yielding bioluminescence. For the latter, the *lux* operon from *Photorhabdus luminescens* [11] or *Vibrio fisceri* [12] is often used, as these bacterial operons encode all components for light emission without the need of adding an exogenous source of a substrate for the luciferase enzyme. This reporter strategy has been used in different bacterial genera that infect a wide range of plant species. As early as the 1980s, the colonization of *Brassica oleracea* leaves by *Xanthomonas campestris* pv. *campestris* (Xcc) was monitored using the first bioluminescent reporter strain [13], followed by another study on the interactions between Xcc and *B. oleracea* [14], *Erwinia amylovora* and apple [15], and *X. oryzae* pv. *oryzae* and rice [16]. At present, bioluminescent reporters are popular for studies on Xcc, either in combination with *B. oleracea* or with Arabidopsis [17–20] and have proven of value in studies on other bacterial pathosystems such as *X. axonopodis* pv. *manihotis* and cassava [21], *X. euvesicitoria* and pepper [21], *X. hortorum* pv. *gardneri* and tomato [22], *Clavibacter michiganensis* and tomato [23], *Agrobacterium tumefaciens* (a.k.a. *Rhizobium radiobacter*) and *Nicotiana benthamiana* [24], *Pseudomonas syringae* pv. *phaseolicola* and bean [25] and Pst and Arabidopsis [26–28].

In general, these studies use bacterial bioluminescence to monitor plant colonization at a macroscopic level, i.e. at the whole plant or whole leaf level. This has revealed highly asymmetric colonization patterns for certain pathogenic bacteria such as the vascular pathogen Xcc [18, 20]. Xcc enters plant leaves via hydathodes, which are organs at the leaf margin involved in the guttation process when root pressure exceeds leaf evaporation. From these hydathodes, Xcc spreads systemically via the connected xylem tissue across the leaf and rosette [18, 20]. Only at a late stage, the leaf mesophyll becomes colonized by Xcc. In contrast, the stomata-invading pathogen Pst has direct access to the entire leaf mesophyll early during the infection. Yet, even for this pathogen, bioluminescence imaging revealed heterogenous patterns of Pst spread in the apoplastic space of Arabidopsis leaves [27]. Notwithstanding the importance of these observations, they are qualitative and thus perform less well when interpreting subtle differences in disease severity (and connected disease susceptibility) using statistics.

Attempts to quantify the bacterial luminescence signal have relied on using luminometers to measure photon emission by bacteria that colonize a single leaf or plant [22]. In some cases, the image processing software ImageJ is used to obtain numerical data from images obtained with ultra-sensitive CCD cameras [21, 28]. The throughput of the former approach is low and the ImageJ macros used are often not well documented [21, 24]. Hence, reproducibility is poor and/or still requires manual preparation of the individual sample images [8]. Clearly, there is a need for a reproducible and objective image analysis tool to monitor microbial spread in mature Arabidopsis leaves. Recently, we have established a procedure to detect bacterial luminescence *in planta* for 126 individual Arabidopsis leaves at once using 40 × 30 cm light-sensitive films [19]. We successfully applied this system to detect differences in disease susceptibility between Arabidopsis accessions and mutants [20]. While this method is more suitable to quantify leaf colonization by the vasculature-infecting Xcc than methods that use counting of colony forming units per leaf area, this method still uses an ordinal qualitive scale based on a luminescence index score that typifies the bacterial spread of the luminescent Xcc in the Arabidopsis leaves in time [19]. The luminescence scores are similar to a disease index scores with their limitations as described. Here, we present an automated luminescence scoring pipeline called ScAnalyzer, which provides an unbiased assessment of leaf chlorosis as a disease symptom and the spread of pathogenic bacteria across the leaf. ScAnalyzer (i) avoids assessments by individual researchers, (ii) provides numerical continuous measure for bacterial spread, and (iii) automates the sample assessment using a Python script to obtain reproducible data.

## Results

### Development of ScAnalyzer, the automated image analysis pipeline to quantify bacterial spread in Arabidopsis leaves

**Fig. 1:**
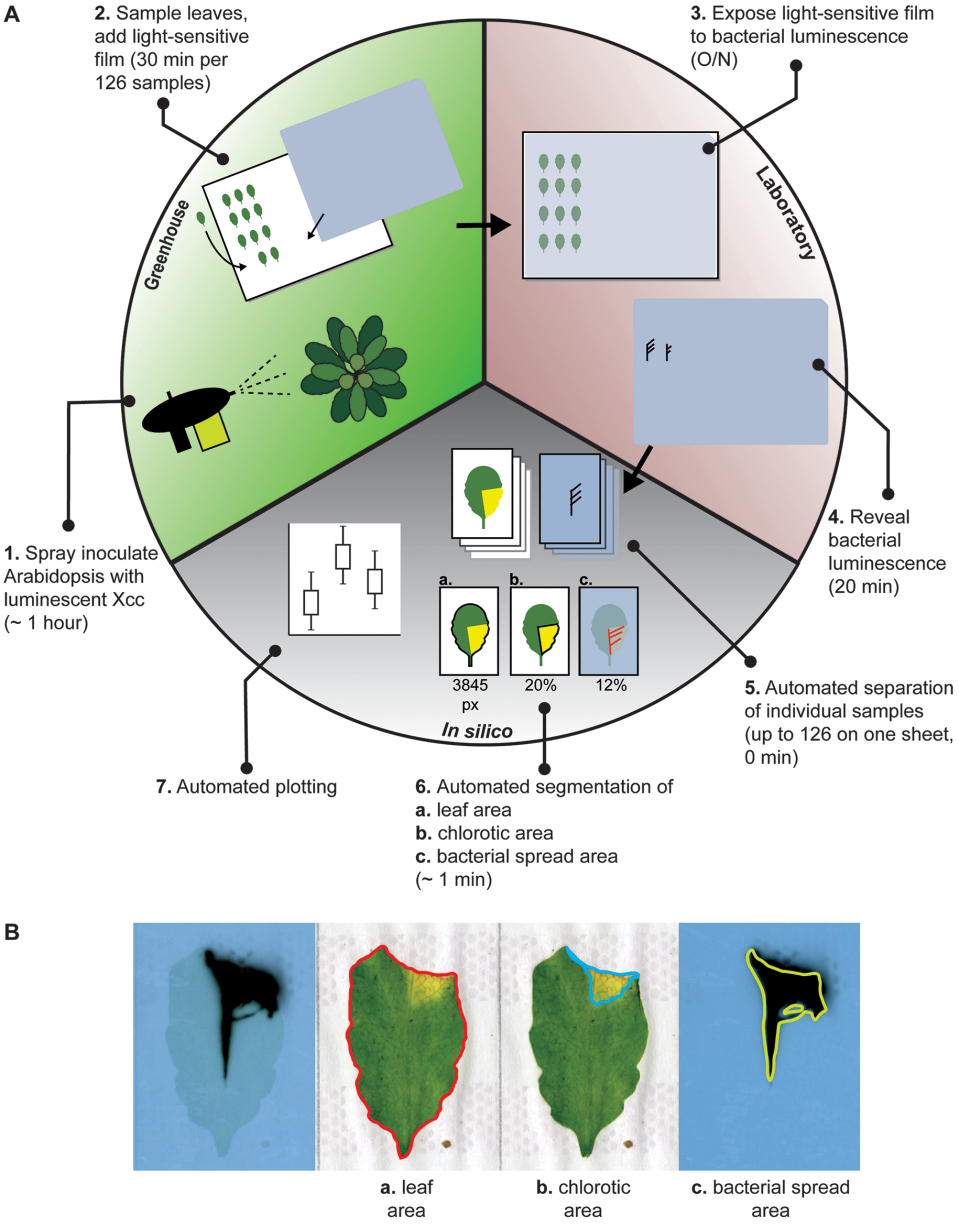
ScAnalyzer: an image analysis pipeline to quantify both chlorosis as proxy for disease symptom severity and bacterial spread. (**A**). Overview of the experimental workflow. Steps are separated by location: greenhouse (green, steps 1 and 2), laboratory (red, steps 3 and 4), and *in silico* (grey, steps 5–7). The greenhouse and laboratory parts are similar to van Hulten et al., (2019) with minor modifications (Table S1). The *in silico* part includes ScAnalyzer and represents a major adaptation of the protocol of van Hulten et al. (2019). (**B**) Example of an infected leaf analyzed with ScAnalyzer pipeline: automated overlay of leaf and detected bacterial luminescence. Based on the thresholds that segment the images, ScAnalyzer extracts the (a) total leaf surface area, while excluding contaminating objects such as soil particles, (b) chlorotic leaf area, and (c) the bacterial spread area, within the leaf boundaries. This example is from a clip-inoculated leaf. The grey dots on the paper are the result of glue roller used to attach the individual leaves on paper

Plant colonization by Xcc was thus far quantified using a method based on defined ordinal luminescence index categories, for which samples were manually evaluated by the researcher [19, 20]. To develop an unbiased and a more automated manner of scoring bacterial spread in Arabidopsis leaves, we developed a streamlined method for leaf sampling and subsequent image analysis (Fig. 1A). After infecting Arabidopsis plants with bacteria tagged with a bioluminescence reporter cassette, we sampled individual leaves from infected plants and organized them in an 18 × 7 grid printed on a 40 × 30 cm (approximately A3) paper sheet. In this way, a total of 126 Arabidopsis leaves can be processed in parallel. The sheets were scanned using a A3 flatbed scanner to standardize the imaging conditions of the leaves between different sampling sheets and experiments. The grid layout of the sampling sheet allowed easy and automated cropping of individual samples using a Python script to analyze each sample individually.

To determine the total and chlorotic leaf area, we defined thresholds in Hue Saturation Value (HSV) color space to first select leaf pixels while removing white background, and potential other objects such as soil particles (Fig. 1B, section a). Another set of thresholds then separate chlorotic (yellow) from healthy (green) leaf area (Fig. 1B, section b). The smallest chlorotic lesions that could be detected in our benchmarking assays were approximately 1.5% of the total leaf area. To quantify the spread of the bacteria using the bioluminescence reporter system, we positioned light-sensitive films (40 × 30 cm sized) on top of the leaf sample sheets and placed them in light-tight cassettes. After overnight exposure, the films were developed and scanned with the same flatbed scanner. Combining the two image files of the leaves and of the bacterial luminescence signal, ScAnalyzer then crops individual leaf samples, and overlays the bacterial luminescence signal on the corresponding leaf scan image. This enabled us to extract the total leaf area colonized by the bacteria for each individual leaf (Fig. 1B, section c), which can be either expressed as total number of pixels colonized per leaf, or corrected for leaf size. To verify image segmentation results, a copy of the image file is saved that highlights the segmented areas on the original images.

The ScAnalyzer pipeline connects each observation to sample metadata (e.g. plant genotype, Xcc genotype) via a pre-defined sample list. ScAnalyzer saves each observation in a *comma separated values* (csv) file directly compatible with statistical analysis software, such as R Tidyverse [29]. After processing all leaves from a single experiment, an R script is invoked to automatically plot the data (including a standard statistical test). The R script automatically detects which grouping variables have multiple levels in the current experiment, and selects the x-axis and faceting variables accordingly. However, manual adaptations to the plot may be necessary depending on the experimental design. An example of an automatically generated plot is shown in Fig S1. All adaptations of the current protocol compared to van Hulten et al. (2019) are shown in Table S1. The pipeline is available from GitHub (https://github.com/MolPlantPathology/ScAnalyzer) as a command-line Python script with installation instructions and a brief user manual.

### ScAnalyzer allows unbiased quantification of disease resistance levels in different Arabidopsis genotypes

**Fig. 2:**
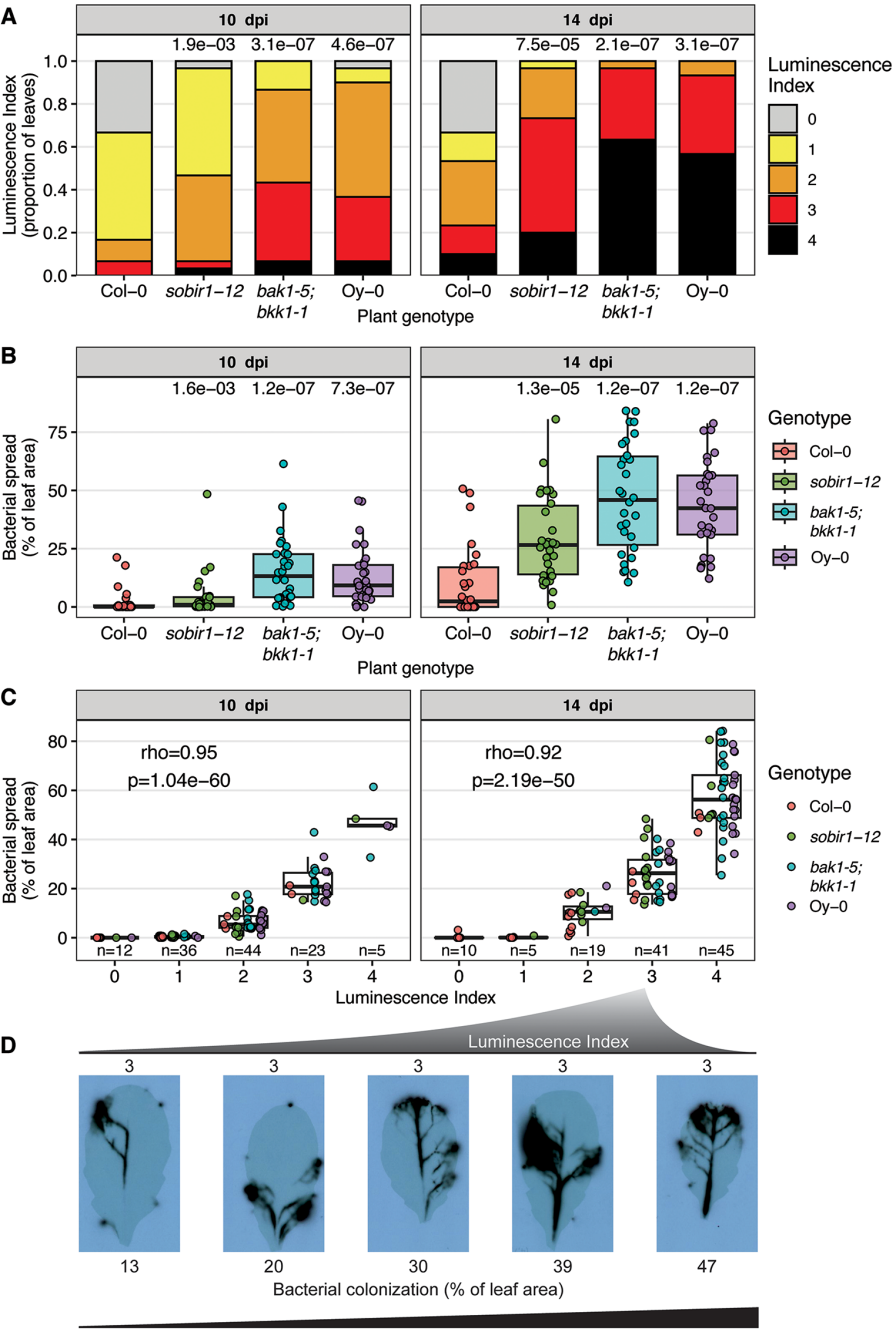
Benchmarking ScAnalyzer against the previous method for assessing disease severity. (**A**) Luminescence index distribution of Xcc *ΔxopAC Tn*7:*lux: mTq2* colonization in Arabidopsis lines. Data from two independent experiments were combined, resulting in a total sample size of *n* = 30 leaves for each treatment. The multiple-testing corrected p-value of pairwise Wilcoxon tests between the mutants and control group Col-0 are reported above the bars. (**B**) Quantification of the bacterial spread using the ScAnalyzer script, of the same leaves displayed in (**A**). (**C**) Correlation between the luminescence index scores and proportion of colonized leaf detected by ScAnalyzer. The number of samples (n) in each luminescence index is indicated above the x-axis. Spearman’s Rho and p-value of the correlation between luminescence index and bacterial colonization is shown in the panel. (**D**) Xcc*-*infected Arabidopsis leaves with luminescence index 3 (indicated above pictures) and bacterial spread ranging from 13–47% of the leaf area (indicated below pictures) reveals subtle differences in bacterial colonization of leaves within luminescence index 3

To benchmark ScAnalyzer, we performed a Xcc disease assay with four Arabidopsis genotypes and accessions known to have different levels of disease resistance to Xcc [20]. Ten and fourteen days post spray inoculation with Xcc, we evaluated leaves of infected Arabidopsis plants using ScAnalyzer. In parallel, we applied the method of van Hulten et al., (2019) and manually assigned an ordinal luminescence index to each individual sampled leaf. Disease scoring based on the luminescence index showed that the immunocompromised mutants *sobir1-12*, *bak1-5;bkk1-1* (both in the Col-0 background) and the hypersusceptible accession Oy-0 showed significantly increased bacterial spread compared to the accession Col-0 at 10 and 14 days post inoculation (dpi) (Fig. 2A, Pairwise Wilcoxon tests, *p* < 0.05), as expected. The same samples were analyzed with the ScAnalyzer pipeline, which gives a continuous score for the bacterial spread per leaf. With ScAnalyzer, the same Arabidopsis lines showed a higher bacterial spread score compared to the control line, Col-0 (Fig. 2B, Pairwise Wilcoxon tests, *p* < 0.05). Both scoring methods showed a strong correlation at 10 and 14 dpi (Fig. 2C, Spearman’s rho = 0.95, *p* < 0.0001). Samples with different luminescence indices (especially in samples with higher luminescence indices 3 or 4) proved to be more accurately scored with ScAnalyzer. For example, several samples with luminescence index 3 show bacterial spread ranging from 13 to 47% of the leaf area (Fig. 2D). In fact, samples with ~ 13% leaf area colonization have also been manually scores to have a luminescence index of 2, and samples with ~ 47% leaf area colonization have been reported to have a luminescence index of 4. Hence, ScAnalyzer strongly improves the resolution and provides a more objective quantification of the degree of bacterial spread when analyzing Arabidopsis plants infected with pathogenic microbes expressing a bioluminescence reporter cassette.

### Bacterial spread precedes the development of leaf chlorosis

**Fig. 3:**
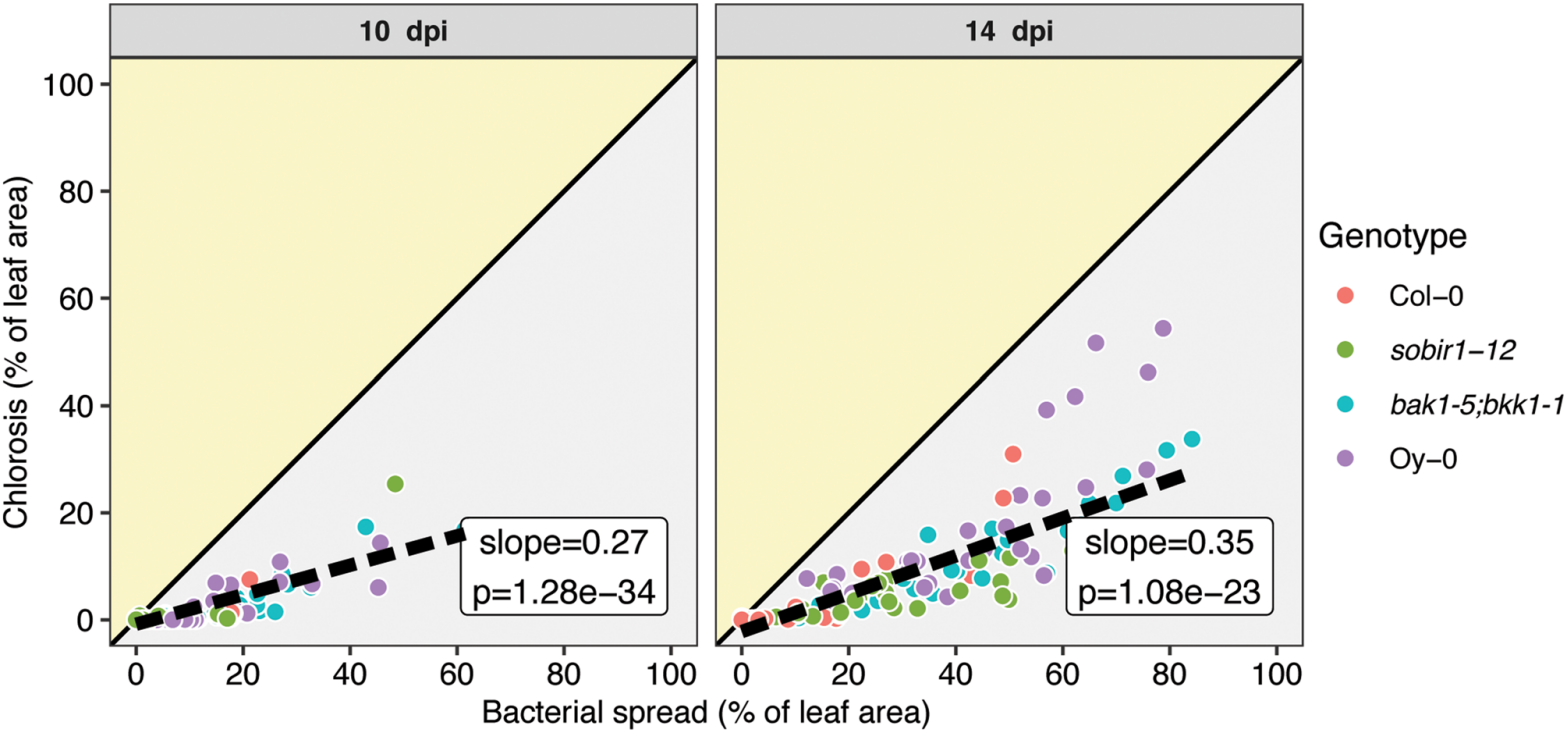
Bacterial spread precedes leaf chlorosis. Correlation between bacterial spread values and chlorotic leaf area reveals that all tested leaves show a higher bacterial spread than chlorotic leaf area, in % of total leaf area. Shaded areas indicate whether the chlorotic area is greater than the area colonized by bacteria (yellow highlight) or vice versa (grey area). The solid black line follows y = x, or, bacterial spread = chlorosis. The dashed black line follows the regression line of the samples, and the inset shows the slope and p value of this line

Besides calculating per leaf the area colonized by bacteria, ScAnalyzer simultaneously determines the chlorotic leaf area. Comparison of these two parameters revealed that for all tested leaves the leaf area colonized by the bacteria was larger than the chlorotic leaf area (Fig. 3). This is substantiated by the slope values of the linear regression lines fitted through the data points, which are less than one (slope = 0.27 and 0.35 at 10 dpi and 14 dpi, respectively). This confirms previously reported observations [13, 20] and indicates that bacterial colonization precedes the development of the typical chlorotic V-shaped lesions associated with black rot disease. However, it is still unclear whether chlorosis is caused by bacteria in situ, or whether chlorosis can occur in distant leaf area without a direct colonization of that same tissue by bacteria.

### ScAnalyzer captures disease symptoms and leaf colonization of stomatal pathogens

**Fig. 4:**
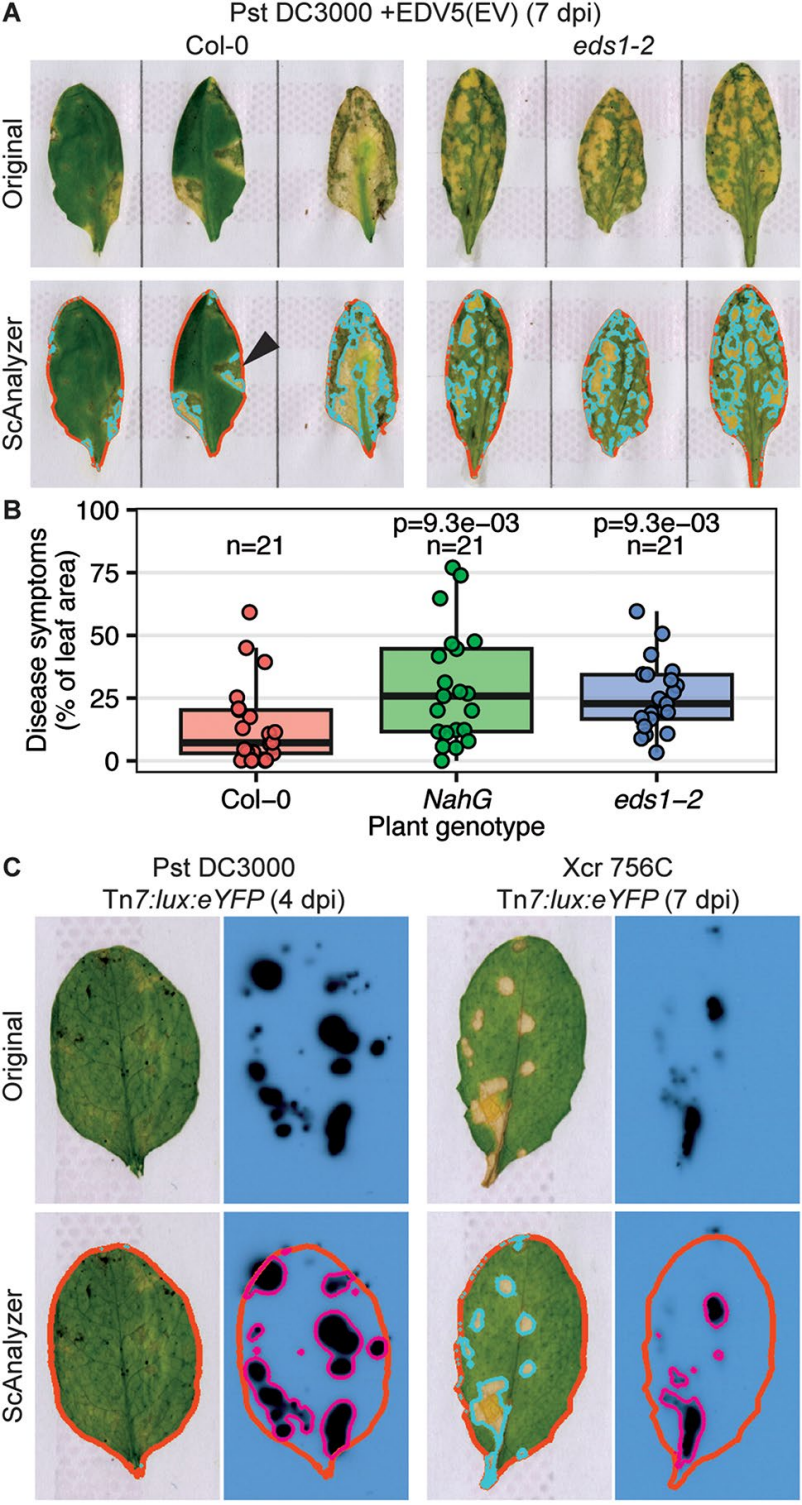
ScAnalyzer captures disease symptoms and bacterial luminescence caused by stomatal pathogens. (**A**) Representative images of Arabidopsis leaves of accession Col-0 and *eds1-2* mutant in Col-0 background infected by Pst DC3000 (carrying empty vector EDV5) at 7 dpi. Top row shows original samples, bottom row shows ScAnalyzer results. Red border: total leaf area. Light-blue border: chlorotic leaf area. Arrow highlights a dark green lesion not detected by ScAnalyzer. (**B**) Quantification of the symptomatic leaf area of leaves of three Arabidopsis lines infected by Pst DC3000 (carrying empty vector EDV5) at 7 dpi. (**C**) Examples of Arabidopsis accession Oy-0 leaves infected with luminescent reporter strains of Pst and Xcr. Top row shows original samples, bottom row shows the ScAnalyzer results. Red border: total leaf area. Light-blue border: chlorotic and necrotic leaf area

To examine whether ScAnalyzer is able to capture the development of chlorotic disease symptoms and leaf colonization by other bacterial pathogens, we performed disease assays with the stomatal pathogen Pst [30] and *X. campestris* pv. *raphani* [31]. Using spray inoculation of a non-luminescent Pst strain, ScAnalyzer was able to detect chlorotic (yellow) and necrotic (white) lesions on Arabidopsis leaves (Fig. 4A). However, dark green lesions were not detected by ScAnalyzer (Fig. 4A, arrow). In addition, compared to the wildtype Col-0 accession, we found a significant increase in the symptomatic leaf area in two hypersusceptible Arabidopsis lines, i.e. in the *NahG* line that does not accumulate the defense hormone salicylic acid [32], and in the immune signaling mutant *eds1-2* [33] (Fig. 4B). In addition, we generated luminescent reporter strains for both Pst and Xcr 756c, and performed spray inoculations. Using these very different bacterial pathogens, the ScAnalyzer pipeline detected spotted patterns of bacterial luminescence across the leaf, reminiscent of stomatal colonization by the bacteria (Fig. 4C). While ScAnalyzer was not able to capture the mild chlorosis in this Pst assay, necrotic and chlorotic lesions caused by Xcr were readily detected (Fig. 4C).

## Discussion

Here, we present the development and benchmarking of a new tool called ScAnalyzer to monitor Xcc disease progression in up to 126 Arabidopsis leaves in parallel. We showed that a simple method based on segmentation of a color image of leaves can detect Xcc disease symptoms. Additionally, we coupled the leaf image to a scan of a film that monitors the presence of the pathogenic bacterium via the signal of a bioluminescence reporter cassette, integrated into the bacterial genome. As a result, ScAnalyzer can quantify both the extent of the disease symptom chlorosis caused by bacterial pathogens and the actual spread of pathogenic bacteria across Arabidopsis leaves. Compared to our previous method to score disease severity and susceptibility in Arabidopsis leaves infected with Xcc [19], ScAnalyzer represents an automated and unbiased method that allows for a more detailed assessment of bacterial spread. While other methods exist to automatically quantify leaf parameters such as shape, colour, and disease symptoms [3–5, 9, 34–37], ScAnalyzer offers the seamless integration of the quantification of microbial luminescence signal in the inspected leaf samples.

ScAnalyzer applies relatively simple image analysis techniques (using pixel color thresholds) to segment the leaf image into healthy and diseased leaf tissue. Importantly, applying color thresholds requires highly standardized imaging conditions of plant material (see Table S1 for optimization steps). Machine learning algorithms, which do not rely on manual threshold selection, have become a popular method for similar tasks, especially in field conditions with less optimized imaging conditions [38, 39]. However, such algorithms require large training sets of data and a certain level of computer science expertise, which are not always readily available.

The ScAnalyzer workflow uses a grid-based sampling, scanning and automated cropping framework which has the potential to be adopted by other researchers in the field of plant pathology. Without much adaptation, it offers a rapid and standardized analysis of Arabidopsis leaves infected with luminescent reporter strains of the model apoplastic pathogen *P. syringae* (Fig. 4) Thus, ScAnalyzer represents an alternative to or complementary method to the more labour-intensive colony count experiments, as suggested by others [27, 40]. With an adjustment of the threshold parameters, the ScAnalyzer pipeline offers the possibility to automatically detect and quantify necrotic lesions caused by *Botrytis cinerea*, although this is not part of the current pipeline. A limitation of the ScAnalyzer pipeline is that it is not designed to detect disease symptoms on plant tissues other than leaves. However, even root-infecting pathogens such as *Fusarium oxysporum* eventually cause chlorotic disease symptoms in Arabidopsis leaves [41], which could be quantified by ScAnalyzer. Beyond the field of plant pathology, the pipeline could be adapted to facilitate the standardized analysis of additional parameters, e.g. leaf size, leaf chlorosis, or anthocyanin accumulation, in leaves of diverse plant species.

The destructive nature of sampling is a potential limitation of the ScAnalyzer workflow, as the same leaves cannot be followed over time. Alternative imaging systems can measure bacterial luminescence signal from whole intact plants or even whole trays of plants without damaging the plants, but these digital phenotyping systems are costly and often not fitted to study plant pathogens in optimal conditions. Nevertheless, they can provide the advantage of tracking disease progression in a non-invasive manner — from initial microbial invasion event to systemic spread across the plant. However, to achieve the resolution and sensitivity for the detection of spatially restricted signals in early infection stages, such as Xcc infections of hydathodes [19, 20] during live-imaging *in planta* in a non-invasive manner, expensive equipment is required (e.g. a light-tight cabinet and an ultra-sensitive CCD camera). Moreover, studying adult Arabidopsis plants over longer periods of time results in overlapping leaves (of the same individual rosettes and between neighboring plants), which hinders accurate scoring. In parallel to the development of such a bioluminescence live-imaging unit for entire plant trays of Arabidopsis [42], we highlight here the advantages for detailed and reliable low-tech imaging of bacterial spread and disease at the level of individual leaves using ScAnalyzer.

## Conclusions

We developed and benchmarked ScAnalyzer, a new image processing tool to monitor disease symptoms and bacterial spread in Arabidopsis leaves.

## Methods

### Bacterial strains and culture conditions

The *Xcc* strain used in this study is a derivative of Xcc strain 8004 [43], carrying a *xopAC* gene deletion [44] and a targeted genomic insertion of a Tn*7:luxCDABE: mTq2* reporter cassette [20, 25], and is listed in Table S2. Xcc was cultured at 28 °C on KADO agar plates (10 g·L^− 1^ sucrose, 8 g·L^− 1^ casamino acids, 4 g·L^− 1^ yeast extract, 2.4 g·L^− 1^ K_2_HPO_4_·3H_2_0, 0.3 g·L^− 1^ MgSO_4_·7H_2_0, and 15 g·L^− 1^ Daishin agar) containing appropriate antibiotics (rifampicin 25 µg·mL^− 1^, gentamycin 10 µg·mL^− 1^). The Pst strains used in this study are derivatives of Pst strain DC3000 [30] and were cultured at 28 °C on Kings B (KB) agar plates (20 g·L^− 1^ proteose peptone, 10 g·L^− 1^ glycerol, 1.966 g·L^− 1^ K_2_HPO_4_·3H_2_0, 1.5 g·L^− 1^ MgSO_4_·7H_2_0, and 15 g·L^− 1^ Daishin agar, pH 7.0). The Xcr strain used in this study is a derivative of Xcr 756c [45], and was grown on KADO agar plates like Xcc. The non-luminescent Pst strain used in Fig. 4A and B carried the plasmid EDV5 without insert (empty vector, EV) [46].

### Bacterial transformations

Bacterial transformations with the Tn*7:lux: eYFP* construct was performed using quadruple parental mating as described [20, 25]. Briefly, recipient cells (Pst DC3000 or Xcr 756c) were co-incubated for 24 h with *E. coli* carrying donor plasmid pRS-Tn7-pnptII::lux-pA1::eYFP [25], and two *E. coli* strains carrying helper plasmids pUX-BF13 [25] and pRK2073. Transformants were selected on LB agarose plates supplemented with gentamycin 10 µg·mL^− 1^ (to select for integration of the luminescence cassette) and nitrofurantoin 50 µg·mL^− 1^ (to eliminate *E. coli* donor and helper strains). To confirm bioluminescence of the transformants, plates with bacterial cultures were inspected using a ChemiDoc MP imager (Bio-Rad).

### Plant cultivation

All *A. thaliana* lines used are listed in Table S2. *A. thaliana* plants were grown as described [19, 20]. Briefly, seeds were stratified in the dark at 4 °C on moist filter paper for 3 days and then sown in 40-pot trays in potting soil (3:17 parts perlite: compost soil, Hol80 zaaigrond Nr1, Jongkind Grond, The Netherlands). The trays were covered with a transparent dome for 5 days to increase the relative humidity and promote equal seed germination. Plants were grown at 22 °C, 70% relative humidity with a short-day light regime (11 h of light, 13 h of darkness).

### Bacterial disease assays

Xcc and Xcr disease assays were performed as described [19, 20]. Briefly, four-week-old plants were used for the disease assays. Xcc was grown from glycerol stock on KADO agar plates at 28 °C for 2 days. Inoculum was prepared by scraping the bacteria from agar plates and dissolving them in 10 mM MgSO_4_. This bacterial solution was then washed by centrifugation (3,000 g, 10 min), decanting the supernatant, and resuspending the pellet in fresh 10 mM MgSO_4_. The inoculum was then diluted to approximately 1 × 10^8^ colony forming units per milliliter (corresponding to OD_600_ = 0.1) and supplemented with 0.0002% Silwett L-77. The inoculum was sprayed on Arabidopis rosettes in a flow cabinet using an airbrush spray-gun. Approximately 25 mL of inoculum was used per 40-pot-tray of plants. The trays were then placed in a growth cabinet (Microclima MC1000, Snijders Labs). To promote the formation of guttation droplets at hydathodes. and thereby natural entry of Xcc, a specific temperature/humidity cycle was used for Xcc disease assays [19]. The Pst disease assays were performed in the same way as the Xcc disease assays described above, except that the plants were covered with a transparent dome to keep the relative humidity above 90% for three days after the inoculation The Pst disease assay in Fig. 4A and B was performed with a bacterial inoculum of OD_600_ = 1.0 supplemented with 0.04% Silwett L-77.

### *In planta* visualization of the bacterial luminescence

Bacterial colonization was monitored at 10 and 14 days post spray inoculation. From each plant, the three most diseased leaves (based on visual disease symptoms) were selected. In absence of disease symptoms, similar leaves were selected (i.e., leaves of the same developmental stage, which were present at the moment of spray inoculation). The selected leaves were attached to a 40 × 30 cm paper sheet using a glue roller. The paper sheet includes a preprinted grid (https://github.com/MolPlantPathology/ScAnalyzer/grid.pdf, actual grid cell dimensions on printed sheet are 22 × 40.5 mm). The grid also contains a predefined header to record experimental metadata (experiment ID, days post inoculation, pathogen ID, date, researcher name). The grid with leaves was then covered with transparent plastic sheets, and placed in a light-tight cassette (30 × 40 cm). A light-sensitive film (Fuji Super RX) was placed on top the sheet and was exposed to the *in planta* bacterial luminescence signal overnight (for approximately 18 h). To ensure proper alignment of the paper sheet with the attached leaves and the film, both were securely positioned using the bottom-left corner as our routine anchor point. The film was developed by submerging it in development solution (AGFA developer/replenisher G150) for 1.5 min, washed in water, and fixed in fixing solution (AGFA, Manual Fixing Bath G354) for 2 min. The black signal of the film caused by bacterial luminescence is indicative of high bacterial densities [19]. To digitize the sheet with leaves and the film, both were scanned in a DIN A3 scanner (Epson A3 Scanner Expression 12000XL) to a JPEG file with the following settings: 300 dpi, high scanning quality, 24-bit color depth, 30 × 40 cm image size. Again, to ensure proper alignment between the sheet and film, the bottom-left corner was used as an anchor point for the scanning.

### ScAnalyzer pipeline description

The ScAnalyzer script was written in Python and uses the OpenCV library for image handling and manipulation [47]. The code is available on GitHub (https://github.com/MolPlantPathology/ScAnalyzer). Briefly, the images of the sheet of leaves and the film are loaded. These images are then separated into 126 cropped image files of 261 × 477 pixels in size based on image coordinates corresponding to the paper grid. Each sub-image file captures a single leaf or the bacterial signal from that corresponding leaf. The image is then analyzed to detect the leaf and chlorotic area using a set of thresholds (in Hue Saturation Value (HSV) color space) to select green and yellow pixels, respectively, followed by contour detection to find the largest shape in the sub-image. Because ScAnalyzer proceeds with only the largest shape in the sub-image, it ensures that other sections of the sub-image that contain green or yellow pixels, such as a piece of soil or small portions neighboring leaves, are not included in the analysis of the current leaf. Within the leaf, chlorotic leaf tissue is separated from healthy leaf tissue detected by separating yellow from green pixels with another set of thresholds in HSV color space. These thresholds were initially determined once by picking HSV colour values of approximately 10 researcher-defined ‘chlorotic’ and ‘healthy’ leaf regions. The corresponding sub-image that contains the bacterial luminescence signal of this leaf is then analyzed similarly to obtain the number of pixels that show bacterial signal above a threshold. Only luminescence signals within the leaf are considered, so any background luminescence signal or luminescence signal from bacteria colonizing the neighboring leaf is not erroneously detected. For each leaf, the following parameters are saved into an .csv file: the total leaf area, total chlorotic area, total bacterial luminescence area. This data is automatically connected to a sample list with metadata provided by the user, which contains grouping information for each sample (plant genotype, pathogen genotype, dpi). The resulting output table is directly compatible for plotting or further analysis in R (v4.2.0) using the packages ggplot2 (v3.4.4) and tidyverse (v2.0.0) [29].

### Statistical analysis

All statistical calculations were performed in R 4.2.0. Details on statistical operations are described in the figure legends and in the results section. All boxplots shown are made with the ‘ggplot2::geom_boxplot()’ function where the middle line represents the median, the upper and lower hinge represent the 75th and 25th percentile respectively, and the upper and lower whisker extend until the largest or smallest value within 1.5 times the interquartile range above the respective hinge. In all boxplots, the all individual data points are plotted as points.

### Electronic supplementary material

Below is the link to the electronic supplementary material.

Supplementary Material 1

## Data Availability

All code and raw images generated during this study are available (https://github.com/MolPlantPathology/ScAnalyzer).
