# Supplementary material for: ScAnalyzer: an image processing tool to monitor plant disease symptoms and pathogen spread in Arabidopsis thaliana leaves
Source: Plant Methods. 2024 May 31;20:80. doi: 10.1186/s13007-024-01213-3 (PMC11141064; doi:10.1186/s13007-024-01213-3)
Supplement: Supplementary file 1 — Supplementary Material 1 [file 13007_2024_1213_MOESM1_ESM.docx]

**Supporting information**

**Figure S1. Example of the automatically generated plot from one experiment.** M65 and M66 are unique identifiers of two independent experimental assays.

**Table S1. Improvements of Arabidopsis – Xcc disease phenotyping in this study, compared to van Hulten *et al.*, (2019)**

| **Step** | **van Hulten *et al*., (2019)** | **This work** | **Purpose** |
| --- | --- | --- | --- |
| Gluing leaves on paper | Random location | In grid | Easy automated cropping of single leaves |
| Scanning | Camera (leaves) and A4 scanner (bacterial presence) | A3 flatbed scanner (both) | Perfect alignment of leaf image and light sensitive film representing bacterial spread |
| Covering leaves | Saran-wrap | Transparent overhead sheet | Obtain image of leaves without reflections |
| Disease assessment | Qualitative,  ordinal classes | Quantitative,  Continuous data | Finer detail of disease severity, more robust statistics |
| Data visualization/analysis | Per experiment, point-and-click software | Automated, open-source software | Faster display of data, unambiguous data analysis |

**Table S2: *Arabidopsis thaliana* lines and bacterial strains used in this study.**

| **Species** | **Genotype** | **Relevant characteristics** | **Identifier (University of Amsterdam** |
| --- | --- | --- | --- |
| *Arabidopsis thaliana* | Col-0 | Model accession | - |
| *Arabidopsis thaliana* | *sobir1-12* | Immunocompromised mutant | ss 1703 |
| *Arabidopsis thaliana* | *bak1-5;bkk1-1 (bb)* | Immunocompromised mutant | ss 1150 |
| *Arabidopsis thaliana* | *eds1-2* | Immunocompromised mutant | ss 768 |
| *Arabidopsis thaliana* | NahG | Salicylic acid accumulation mutant | ss 541 |
| *Arabidopsis thaliana* | Oy-0 | Hypersusceptible accession | ss 1750 |
| *Xanthomonas campestris* pv. *campestris*, strain 8004 | *ΔxopAC* *Tn*7*:lux:mTq2* | Bioluminescent and fluorescent reporter strain lacking type III effector XopAC | bglFP 6920 |
| *Xanthomonas campestris* pv. *raphani*, strain 756c | *Tn*7*:lux:eYFP* | Bioluminescent and fluorescent reporter strain | bglFP 6708 |
| *Pseudomonas syringae* pv. *tomato*, strain DC3000 | EDV5(EV) | Carrying an empty expression vector. | bglFP 8324 |
| *Pseudomonas syringae* pv. *tomato*, strain DC3000 | *Tn*7*:lux:eYFP* | Bioluminescent and fluorescent reporter strain | bglFP 6699 |
